# Supplementary material for: Contrasting social and non-social sources of predictability in human mobility
Source: Nat Commun. 2022 Apr 8;13:1922. doi: 10.1038/s41467-022-29592-y (PMC8993859; doi:10.1038/s41467-022-29592-y)
Supplement: Supplementary file 1 — Supplementary Information [file 41467_2022_29592_MOESM1_ESM.pdf]

Supporting Information

Contrasting social and non-social sources of predictability in human mobility

Zexun Chen, Sean Kelty, Alexandre G. Evsukoff, Brooke Foucault Welles, James P. Bagrow, Ronaldo Menezes, and Gourab Ghoshal

CONTENTS

S1. Data S-3
S1.1. Dataset collection S-3
S1.2. Mobility Statistics S-4
S1.3. Pre-processing and estimator convergence S-5

S2. Ego-alter network construction in LBSN S-7
S2.1. Quality control of co-locators S-7
S2.2. Choice of the number of top alters S-8
S2.3. Summary of filtered networks S-10

S3. Information contained in alters S-10

S4. Extrapolating Cross-Predictability S-15

S5. Spatial correlation analysis S-16
S5.1. Correlation in LBSN S-16
S5.2. Correlation in CDR S-23

S6. Time lag effect S-25
S6.1. Sensitivity in LBSN S-25
S6.2. Sensitivity in CDR S-27

S7. Robustness and Controls Analysis S-27
S7.1. Robustness of the threshold for the minimum number of check-ins S-27
S7.2. Robustness of temporal windows in colocation network construction S-28

S7.3. Robustness to excluding "non-informative" alters S-29
S7.4. Location-overlap preserving controls S-31

References S-32

S1. DATA

S1.1. Dataset collection

- *BrightKite* is a LBSN service provider that allowed registered users to connect with their existing social ties and also meet new people based on the places that they go. Once a user "checked in" at a place, they could post notes and photos to a location and other users could comment on those posts. The social relationship network was collected using their public API. Dataset link: https://snap.stanford.edu/data/loc-brightkite.html

- *Gowalla* is a LBSN website where users share their locations by checking-in. In early versions of the service, users would occasionally receive a virtual "Item" as a bonus upon checking in, and these items could be swapped or dropped at other spots. Users became "Founders" of a spot by dropping an item there. This incentivises users to create new check-ins, not necessarily to check-in consistently at frequently visited locations. The social relationship network is undirected and was collected using their public API. Dataset link: https://snap.stanford.edu/data/loc-gowalla.html

- *Weeplaces* - This is collected from Weeplaces and integrated with the APIs of other LBSN services, e.g., Facebook Places, Foursquare, and Gowalla. Users can login Weeplaces using their LBSN accounts and connect with their social ties in the same LBSN who have also used this application. Weeplaces visualizes your check-ins on a map. Unlike Gowalla, there is no direct incentive in Weeplaces to alter one's visitation habits or check-ins, so there should be a more accurate representation of a regular person's mobility patterns. Dataset link: https://www.yongliu.org/datasets/

- *Mobile Phone* - This is a Call Detail Record (CDR) dataset provided by one of the largest telephone companies in Brazil, and it was collected during 2014 (time period is between Jan 9, 2014 and Jun 27, 2014.), in the Rio de Janeiro, Brazil, Metropolitan Area (RJMA). Data were collected from the outgoing voice calls of 2.9 million mobile phone users through all 1835 antennas. There are 22,116,252 call records by 35,338 users. User location is related to the nearest tower, such that tower coverage can be approximated using Voronoi polygons. All user data in anonymized

preserving privacy, but it is possible to identify calls made/received by users of the same operator. Mobility results extracted from the dataset is available at https://dataverse.harvard.edu/dataverse/MRRJ.

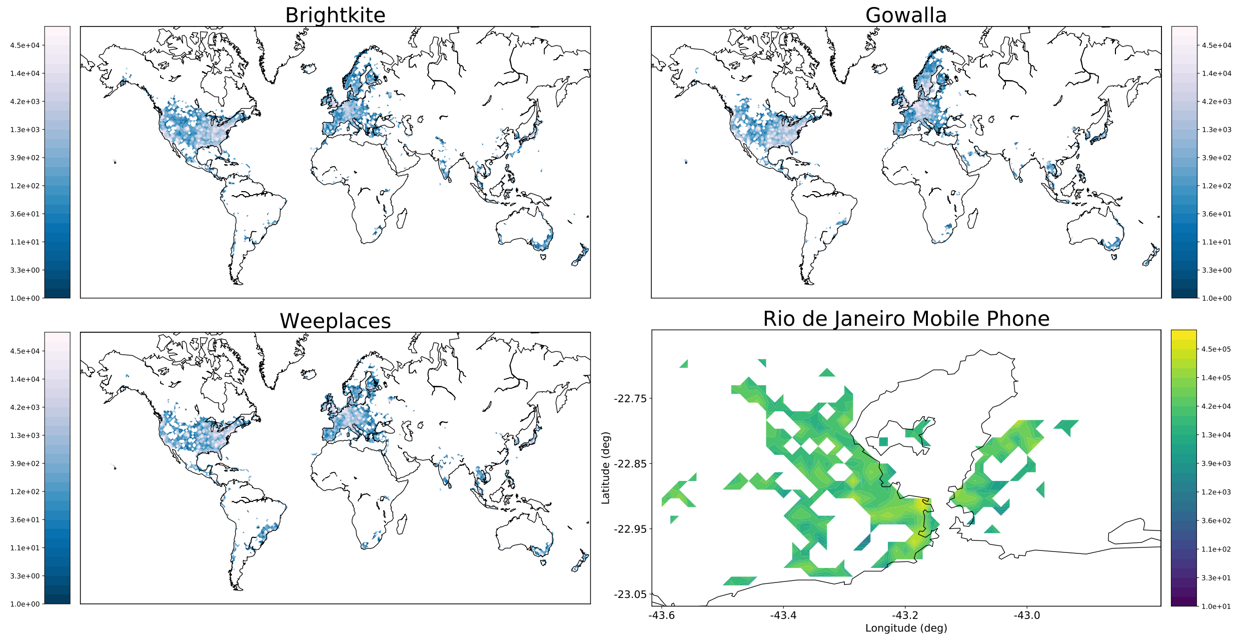

FIG. S1. **Check-in Maps of Gowalla, BrightKite, Weeplaces, and Mobile Phone Dataset.** The colorbar signifies the number of check-ins within a 50km radius for the LBSNs, and shows the highest coverage in North America and Western Europe. The colorbar for the Rio de Janeiro, Brazil uses a different scale due to the fact we are dealing with city-scale values. Worldmaps generated using the Matplotlib Basemap Toolkit [1].

S1.2. Mobility Statistics

The number of unique visited locations, the distribution of jump lengths and the radii of gyration for all pre-processed datasets are shown in Figure S2. The latter two quantities are qualitatively identical among the three datasets and consistent with other sources. The distribution of jump lengths resembles a power law distribution, and the tail of the distribution of the radius of gyration closely represents a truncated power law. The distributions of the number of distinct locations illuminate characteristic differences between the datasets. Users of Gowalla and Weeplaces are more likely to to visit many

locations, while users of BrightKite check-in at very few distinct locations. Mobile Phone users also visit few distinct locations due to the spatial granularity of cell-tower coverage.

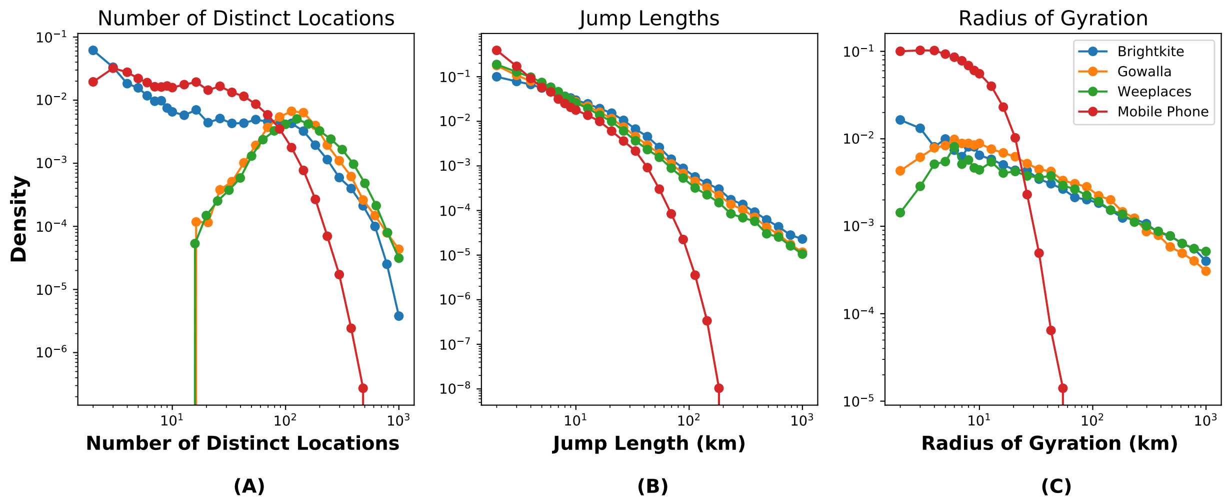

FIG. S2. **Statistical distributions of selected mobility quantities (A)** Distribution of total distinct locations visited by all users in each dataset. **(B)** Distribution of Jump Lengths of all check-ins of users in the datasets **(C)** Histogram of Radii of Gyration for all users in the datasets

S1.3. Pre-processing and estimator convergence

Because the LBSN data is finite and fairly sparse, we need to understand how well the entropy estimators saturate and set thresholds for our data for robustness. We establish a 150 check-in threshold that yields a sufficient amount of data to analyze the both the individual user entropy and cross entropy, which is demonstrated in Figure S3. The user entropy (Figure S3A) for all datasets is shown to stabilize well into their own trajectory. In the case of the cross entropy, we apply the condition that alters should check in at least 150 times before the final check-in of the ego, to assert that enough data is present to estimate the cross entropy. To examine the saturation of the cross-entropy estimator (Figure S3B), we partition an ego's trajectory into two portions and compare the variances of the cross-entropy values of the two portions. We use a 50-50 split and an 80-20 split to show how well the cross entropy saturates in the final portion of the data compared to the previous. The 80-20 split was chosen with the 150 check-in minimum in mind, so the final 20% of the data would have at least 30 check-ins for which one could reasonably

calculate a variance. The two partitions show that as the number of check-ins increase, the variance in the latter portion relative to the earlier portion is much smaller, with the 80-20 split indicating a higher difference in variances, indicating that the cross entropy stabilizes with time and a 150 check-in threshold is sufficient to realize this convergence. After pre-processing, the summary statistics are shown in Table S1.

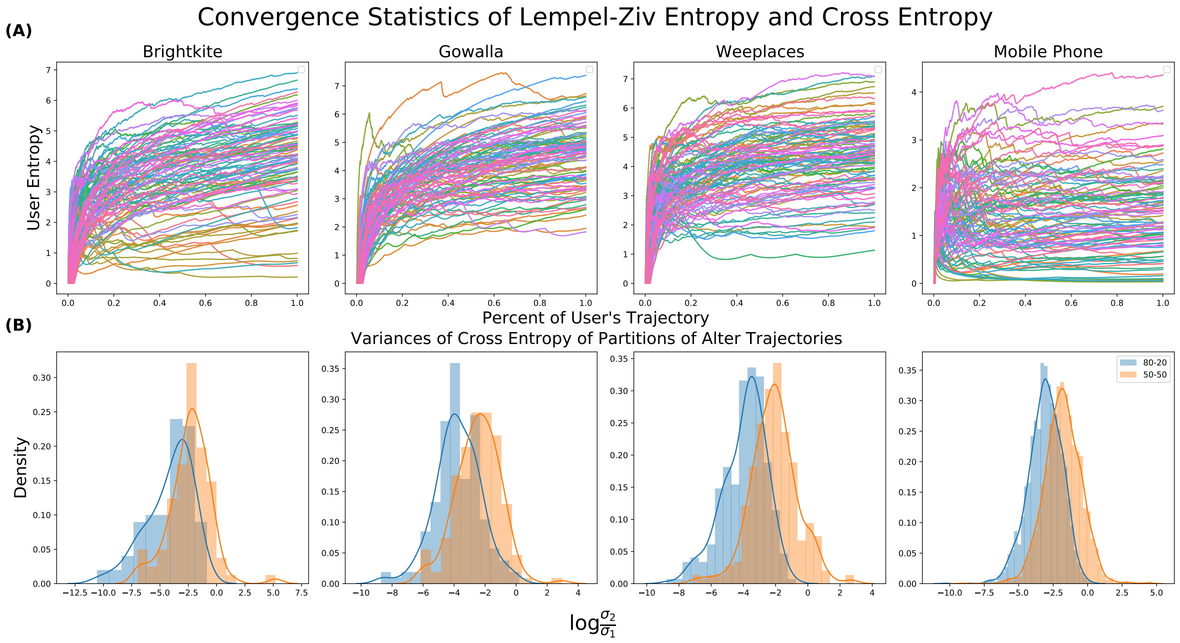

FIG. S3. **(A)** Entropy rate as a function of a percentage of the ego's trajectory. **(B)**, The log ratio of the standard deviations of the cross entropy of the end and beginning partitions show much lower variability in the latter end of the trajectory, signifying a leveling-off of the cross entropy estimator.

TABLE S1: **The summary of three pre-processed datasets**.

Dataset	Total Check-ins/Call Records	Users	Distinct Placeid
BrightKite	3,513,895	6,132	510,308
Gowalla	3,466,392	9,937	850,094
Weeplaces	7,049,037	11,533	924,666
Mobile Phone	1,382,626	4,415	1,816

S2. EGO-ALTER NETWORK CONSTRUCTION IN LBSN

In each of the LBSNs we use, there exist both a location check-in network and a social network, so we can use the social network to compare against a proxy network. With the check-in network, we can form an artificial social network by assigning connections to users that check in at the same place at the same time. We took a colocation as two users checking in at the same place-id within an hour starting on the hour, e.g. 8:00 - 9:00 (The choice of the 1-HR bin for colocation was examined, and the details can be found in Section S7.2). We assume that users that co-locate more often contain more predictive information about each other's whereabouts, so we rank users' social relationship based on the number of times they co-locate, both in the social relationship and colocation network. Because two people that co-locate are not necessarily social ties, we use the term "ego" to describe users whose mobility data we are trying to predict by the location history of their "alters" (non-social colocators and social ties).

S2.1. Quality control of co-locators

We enforce the reasonable constraint that egos and alters (whether social or non-social ties) must co-locate at least more than once across the temporal history of the datasets. Correspondingly, we discard all ego-alter pairs that either do not co-locate or co-locate only once and keep the rest. We note that if one were to make a random guess on an ego's next location, at worst that is equivalent to $1/n_{ego}$ where n_{ego} is the number of unique locations in their historical trajectory. The corresponding information provided due to this random guessing is $\log_2(n_{ego})$ bits.

As an example of such users in our database, consider the following: an ego A, and their alter B in the Weeplaces dataset. The number of unique locations visited by A is $n_A = 254$ and the total check-ins are $N_A = 524$. For B the corresponding numbers are $n_B = 250$ and $N_B = 544$. The check-in time period for A is [2009-03-15 01:16:25, 2010-10-18 18:38:33] while that for B is [2009-10-29 02:55:32, 2010-10-21 23:54:30]. Correspondingly $1/n_A = 0.003937$ and $\log_2(254) = 7.9887$. Although B checked in 541 times while A was active, A only checked in 384 times during the period that B was active. That is to say, the whole check-in sequence of B contributes nothing to the first $524 - 384 = 140$ check-ins of

A. Mathematically speaking, 140 zeros at the beginning of the sum in the denominator of Eq. (4) result in a relatively large $\hat{S}_{A|B}$. In this example, $\hat{S}_{A|B} = 8.4280 > 7.9887 = \log_2(254)$, therefore alter B's trajectory provides no more information on A than simple random guessing based on A's location history.

For purposes of statistical significance we partition the alter set into those who provide information on an ego equivalent to random guessing, that is $\log_2(n_{ego})$ bits, and those whose cross-entropy is less than $\log_2(n_{ego})$ bits. We term the former "**non-informative**" and the latter "**informative**". In the manuscript, we present results derived from the "informative" alters, in Sec. S7.3 we show the results when also including the "non-informative" alters.

S2.2. Choice of the number of top alters

We make the reasonable assumption that alters with a higher frequency of colocations provide more information than those with lower frequency. We measure the average number of colocations per rank of the datasets in Figure S4 finding that in the LBSN datasets, beyond roughly 10 alters, the number of colocations saturates at fewer than around 5 meetups, making the potential influence due to colocation insignificant. In Mobile Phone dataset this occurs for around 20 alters, although the curve begins to flatten near 10. For a fair comparison across all datasets, we therefore focus on the contribution from the top ten alters. To validate our hypothesis, we plot the cross-entropy distributions of rank-5 (middle) and rank-10 (low) alters for both networks in all datasets, finding that on average the entropies increased with lower rank (Figure S5).

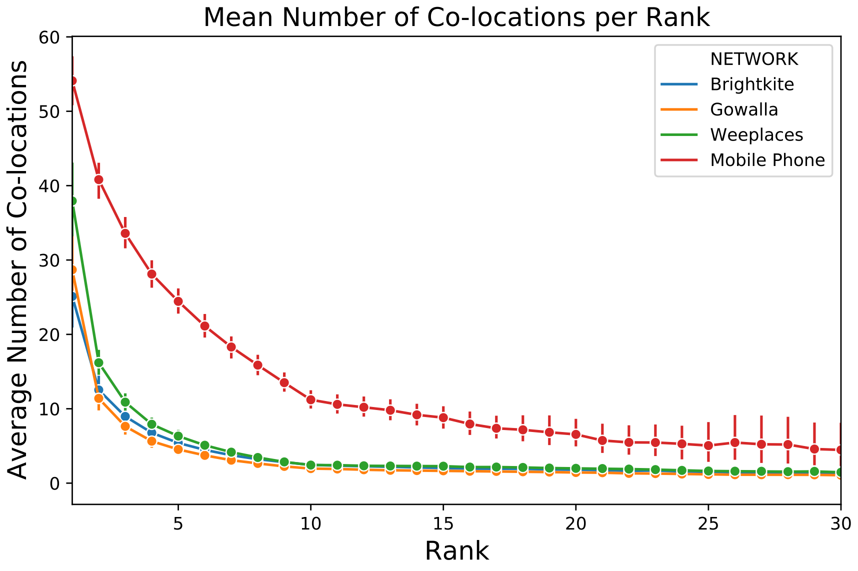

FIG. S4. **Average number of colocations per rank for all datasets**

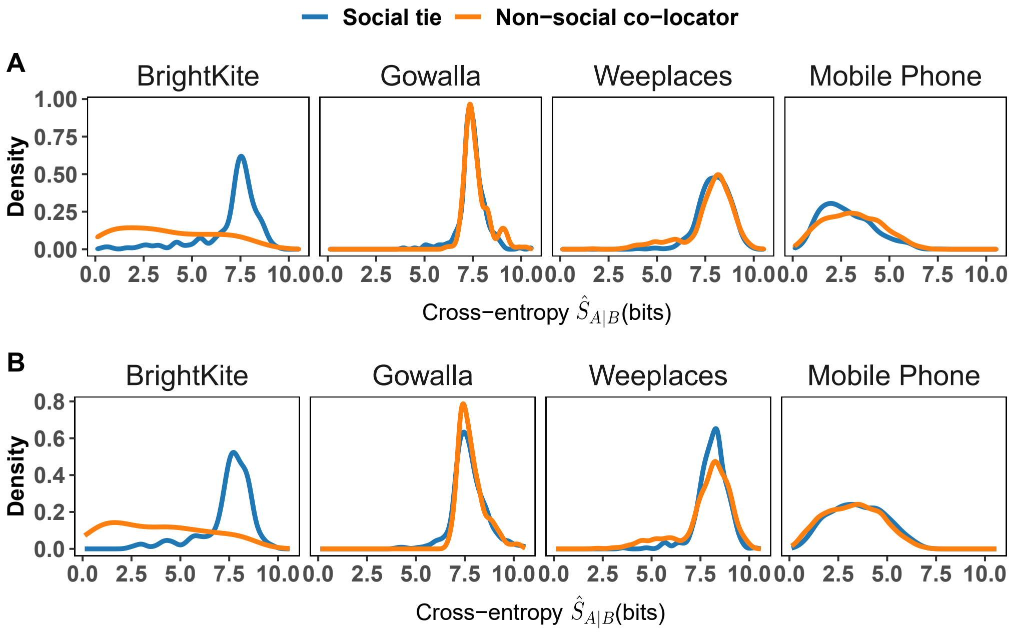

FIG. S5. **Information contained in rank-5 and rank-10 alter. (A)** Cross-entropy of ego with their rank-5 social tie and rank-5 non-social colocator. **(B)** Cross-entropy of ego with their rank-10 social tie and rank-10 non-social colocator.

S2.3. Summary of filtered networks

The summary statistics of the resulting networks are shown in Table S2.

TABLE S2: **The summary of filtered networks**. The number of egos who have at least ten alters in both non-social colocation and social-networks. The common networks are those which share the same egos with at least ten alters in each network.

Dataset	Non-social colocation network		Social network		Common-Ego networks	
	ego	ego-alter pair	ego	ego-alter pair	ego	ego-alter pair
BrightKite	122	2,684	187	4,460	33	330
Gowalla	192	9,332	349	7,681	97	970
Weeplaces	665	21,741	401	8,042	199	1,990
Mobile Phone	3347	198, 395	483	7,052	483	9,660

S3. INFORMATION CONTAINED IN ALTERS

Figure S6, Figure S7, and Figure S8 show the information-theoretic analysis of BrightKite, Gowalla, and Mobile Phone, respectively

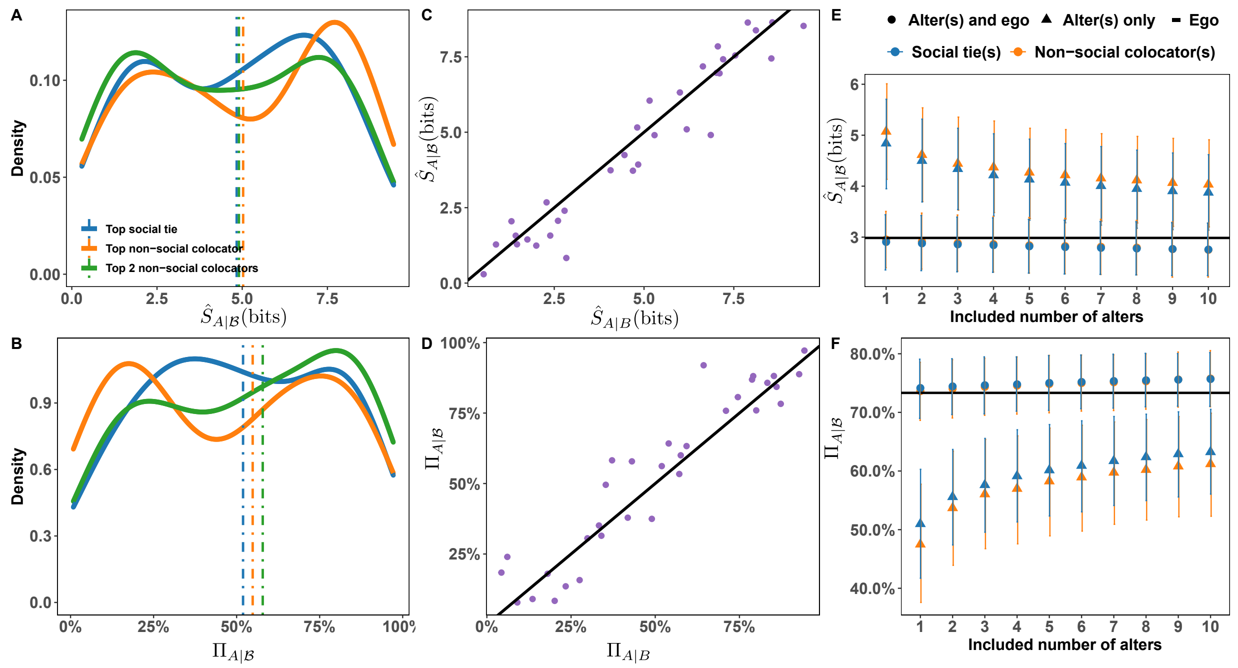

FIG. S6. **The cross-entropy and predictability provided by social ties and non-social colocators in BrightKite**. **A** Distributions of $\hat{S}_{A|\mathcal{B}}$ for the rank-1 social tie (median 4.84 bits), non-social colocator (median 5.03 bits), and $\hat{S}_{A|\mathcal{B}}$ for the top-2 non-social colocators (median 4.90 bits) in **B** The corresponding $\Pi_{A|\mathcal{B}}$ for the social (median 51.94%), and non-social colocators (median 54.84%), and $\Pi_{A|\mathcal{B}}$ for the top-2 non-social colocators (median 57.86%). **C** $\hat{S}_{A|\mathcal{B}}$ encoded in the top-social tie as a function of $\hat{S}_{A|\mathcal{B}}$ for the top-2 non-social colocators. Each point corresponds to a single ego and the solid line denotes $y = x$. **D** As in panel **C** but with predictability instead of cross-entropy. **E, F** $\hat{S}_{A|\mathcal{B}}$ and $\Pi_{A|\mathcal{B}}$ after accumulating the top-ten social alters and non-social colocators. Horizontal lines denote the average entropy (2.98 bits) of egos and their self-predictability (73.33%). Shapes indicate whether the past trajectory of the ego was included in the sequence (circles) or excluded (triangles). Error bars denote 95% CI.

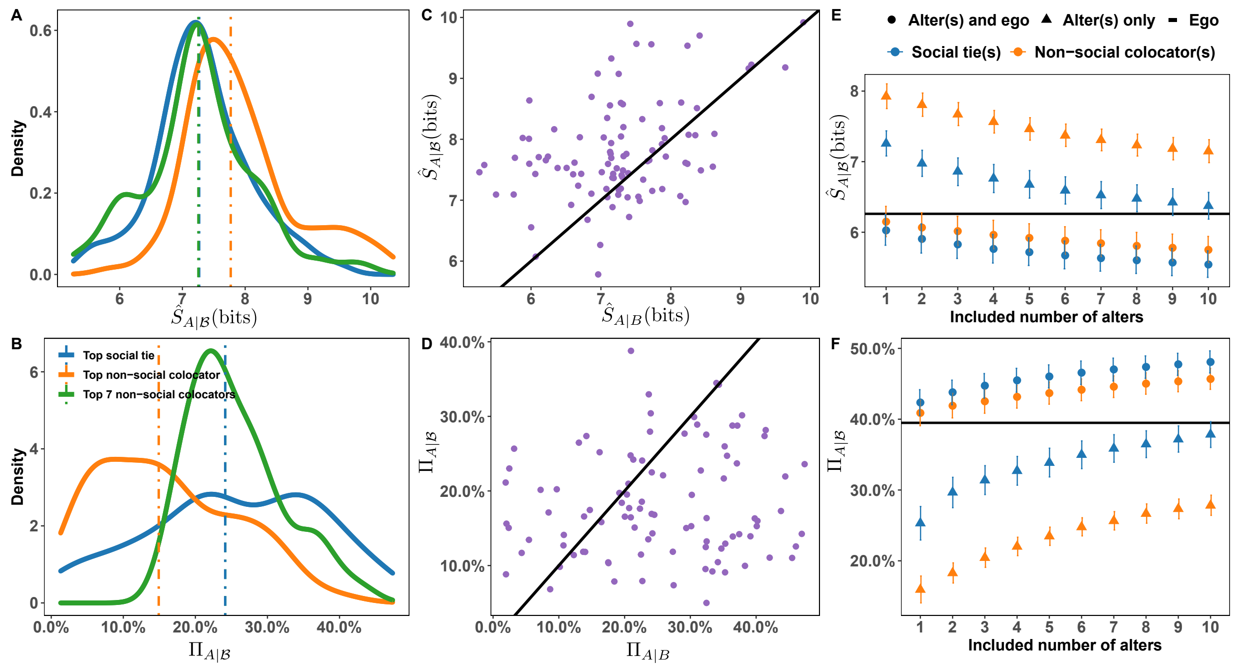

FIG. S7. **The cross-entropy and predictability provided by social ties and non-social colocators in Gowalla**. **A** Distributions of $\hat{S}_{A|\mathcal{B}}$ for the rank-1 social tie (median 7.27 bits), non-social colocator (median 7.77 bits), and $\hat{S}_{A|\mathcal{B}}$ for the top-7 non-social colocators (median 7.26 bits) in **B** The corresponding $\Pi_{A|\mathcal{B}}$ for the social (median 24.14%), and non-social colocators (median 14.92%), and $\Pi_{A|\mathcal{B}}$ for the top-7 non-social colocators (median 24.14%). **C** $\hat{S}_{A|\mathcal{B}}$ encoded in the top-social tie as a function of $\hat{S}_{A|\mathcal{B}}$ for the top-7 non-social colocators. Each point corresponds to a single ego and the solid line denotes $y = x$. **D** As in panel **C** but with predictability instead of cross-entropy. **E, F** $\hat{S}_{A|\mathcal{B}}$ and $\Pi_{A|\mathcal{B}}$ after accumulating the top-ten social alters and non-social colocators. Horizontal lines denote the average entropy (6.26 bits) of egos and their self-predictability (39.49%). Shapes indicate whether the past trajectory of the ego was included in the sequence (circles) or excluded (triangles). Error bars denote 95% CI.

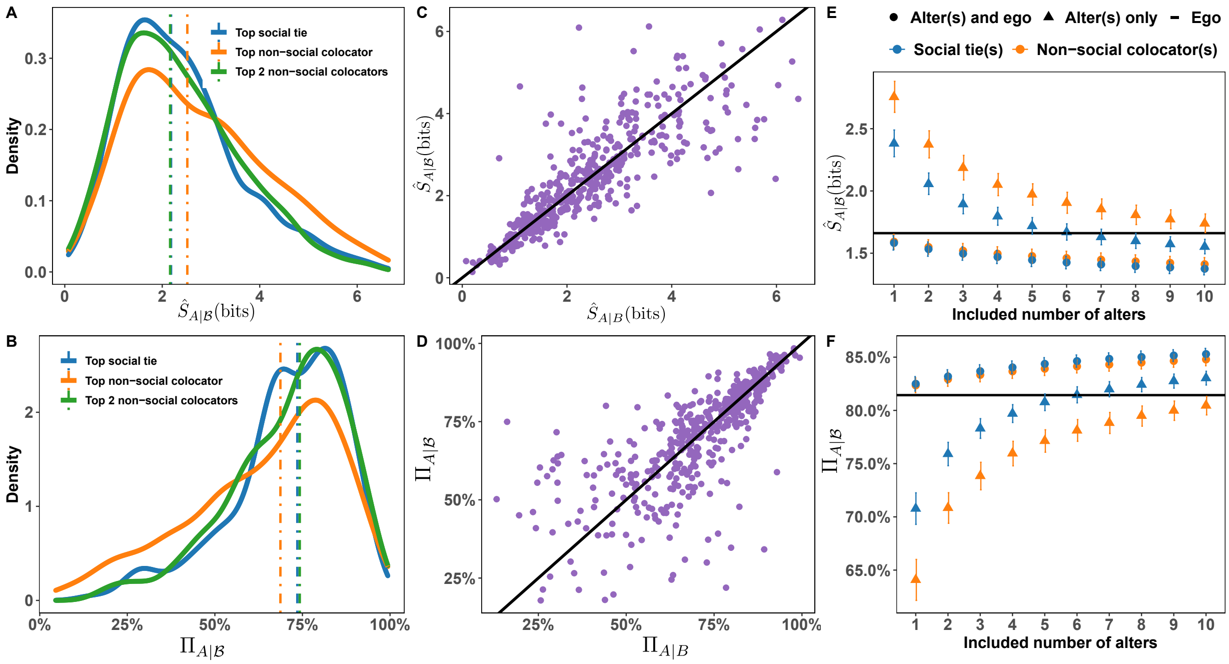

FIG. S8. **The cross-entropy and predictability provided by social ties (30-reciprocal-call ties) and non-social colocators (no-call-history colocators) in Mobile Phone dataset.** **A** Distributions of $\hat{S}_{A|\mathcal{B}}$ for the rank-1 social tie (median 2.38 bits), non-social colocator (median 2.75 bits), and $\hat{S}_{A|\mathcal{B}}$ for the top-2 non-social colocators (median 2.37 bits) in **B** The corresponding $\Pi_{A|\mathcal{B}}$ for the social (median 70.78%), and non-social colocators (median 64.09%), and $\Pi_{A|\mathcal{B}}$ for the top-2 non-social colocators (median 70.85%). **C** $\hat{S}_{A|\mathcal{B}}$ encoded in the top-social tie as a function of $\hat{S}_{A|\mathcal{B}}$ for the top-2 non-social colocators. Each point corresponds to a single ego and the solid line denotes $y = x$. **D** As in panel **C** but with predictability instead of cross-entropy. **E, F** $\hat{S}_{A|\mathcal{B}}$ and $\Pi_{A|\mathcal{B}}$ after accumulating the top-ten social alters and non-social colocators. Horizontal lines denote the average entropy (1.66 bits) of egos and their self-predictability (81.43%). Shapes indicate whether the past trajectory of the ego was included in the sequence (circles) or excluded (triangles). Error bars denote 95% CI.

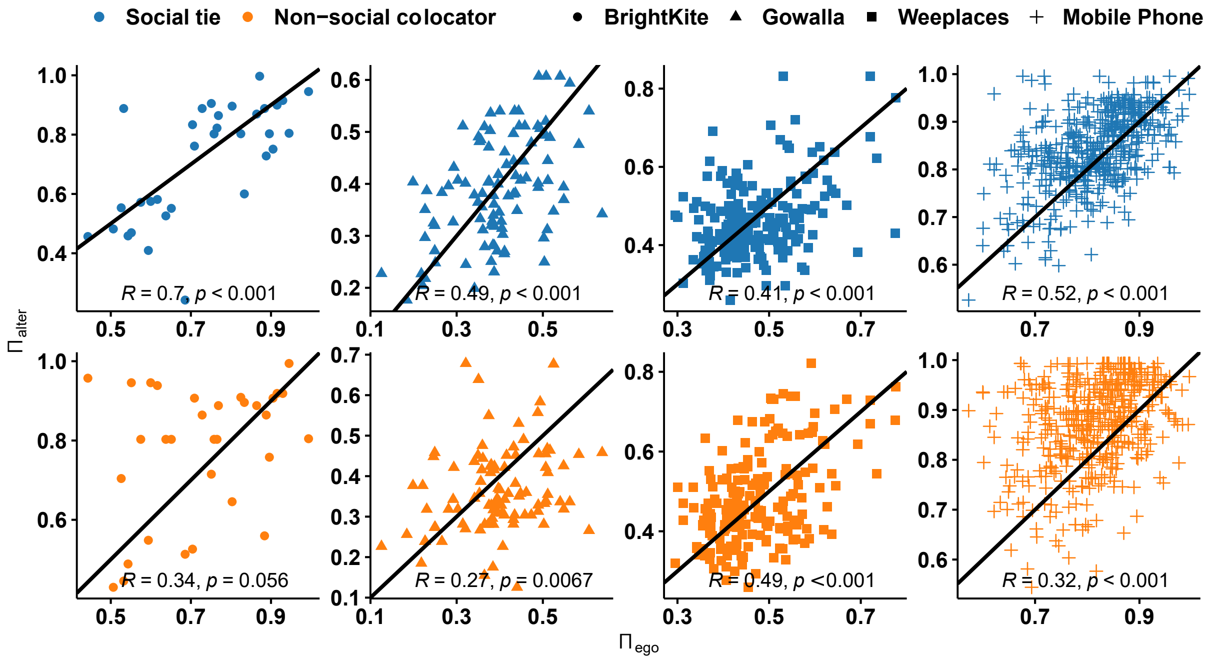

FIG. S9. **Homophily in predictability**. Scatterplot comparing the predictabilities of egos to their rank-1 alters. All egos are those who have at least both 10 social ties and 10 non-social colocators. The black solid lines in each subplot are $y = x$.

S4. EXTRAPOLATING CROSS-PREDICTABILITY

We've chosen the top 10 alters in determining the cumulative mobility information flow between the alters' respective egos. We extrapolate these results by fitting a saturating function to our data, to determine the potential information flow in the limit of infinite alters (or more realistically around 150 alters, the maximum number of social ties a given person can reasonably have). The saturating function used is

$$\Pi(i) = \Pi_\infty + \frac{\beta_0}{\beta_1 + i} \tag{S1}$$

where i is the number of top i included alters. A χ^2 minimization of the means and their errors using the BFGS algorithm was used to determine the most likely parameters. A 95% confidence interval of the parameters was determined using a t-test with 10 alters -3 parameters $=$ 7 degrees of freedom. Results for fitting the cumulative cross-predictability, with and without including the ego's past trajectory, can be found in Table S3 and Table S4.

TABLE S3: **Parameters for saturating function of the cumulative cross-predictability** $\Pi(i)$

Dataset & Network	BrightKite		Gowalla		Weeplaces		Brazil CDR	
	Soc	Coloc	Soc	Coloc	Soc	Coloc	Soc	Coloc
Π_∞	0.6699 ± .003313	0.6329 ± .00504	.4319 ± 0.005082	.3897 ± .003186	0.4431 ± .001039	0.3979 ± 0.003427	.8568 ± 3.38 E-4	.8406 ± .001169
β_0	-0.4629 ± .03817	-0.2980 ± .0445	-.7427 ± .07489	-1.881 ± .0747	-0.7792 ± 0.01240	-1.616 ± .0662	-.2927 ± .003490	-.4143 ± .01124
β_1	1.937 ± .2011	.9096 ± .25089	3.224 ± .3323	7.135 ± .2237	2.083 ± 0.04045	5.307 ± .1865	.9694 ± .02101	1.083 ± .04974

TABLE S4: **Parameters for saturating function of the cumulative cross-predictability $\Pi(i)$ including the ego's past trajectory**

Dataset & Network	BrightKite		Gowalla		Weeplaces		Brazil CDR	
	Soc	Coloc	Soc	Coloc	Soc	Coloc	Soc	Coloc
Π_∞	0.7844 ± .002371	0.7759 ± .00149	.5203 ± 0.001491	.5182 ± .002374	0.5670 ± 7.04 E-4	0.5625 ± 0.001727	.8710 ± 2.00 E-4	.8657 ± 4.79 E-4
β_0	-0.6886 ± .09488	-0.3426 ± .09208	-.6207 ± .02950	-1.280 ± .07557	-0.5742 ± 0.01527	-0.9300 ± .05103	-.2689 ± .003734	-.2857 ± .009870
β_1	15.08 ± 1.363	7.549 ± 0.0046	5.458 ± .2198	10.77 ± .459	6.156 ± 0.1353	10.34 ± 0.395	4.848 ± .0599	5.801 ± .1662

S5. SPATIAL CORRELATION ANALYSIS

S5.1. Correlation in LBSN

We plot the correlation between the cumulative cross-predictability and the CODLR in both types of networks as one progressively adds alters from rank-1 to rank -10 in Figure S14 and Figure S15 for the Weeplaces dataset. While including a single alter yields a Pearson correlation coefficient $R = 0.13$ in colocation network and $R = 0.27$ in social network, the correlation increases as one progressively adds more alters saturating at $R = 0.67$ and $R = 0.66$ in colocation network and social network, respectively. We can also the same trend in both BrightKite (See Figure S10 and Figure S11, and Gowalla ((See Figure S12 and Figure S13) dataset.

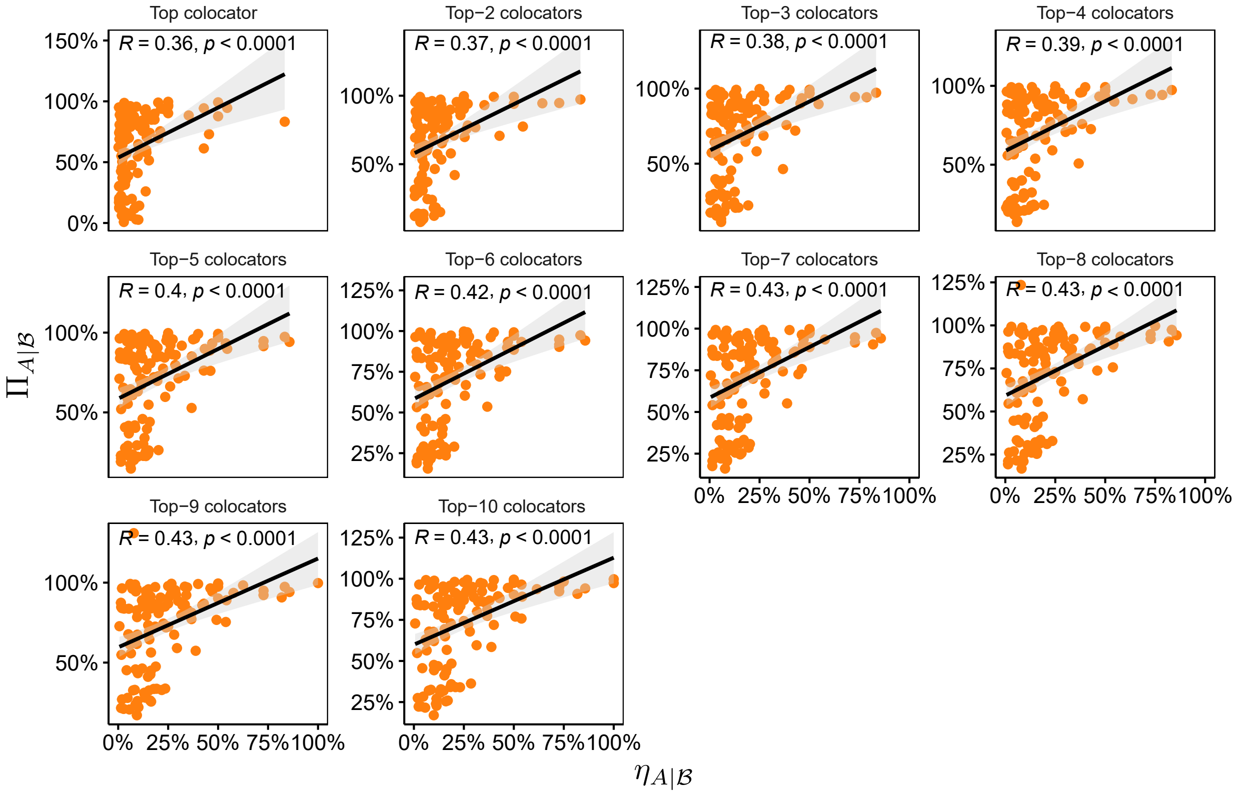

FIG. S10. **CODLR vs cumulative cross-predictability for non-social ties in BrightKite.** R is Pearson's correlation coefficient and p is p-value. The solid black lines are linear regression lines.

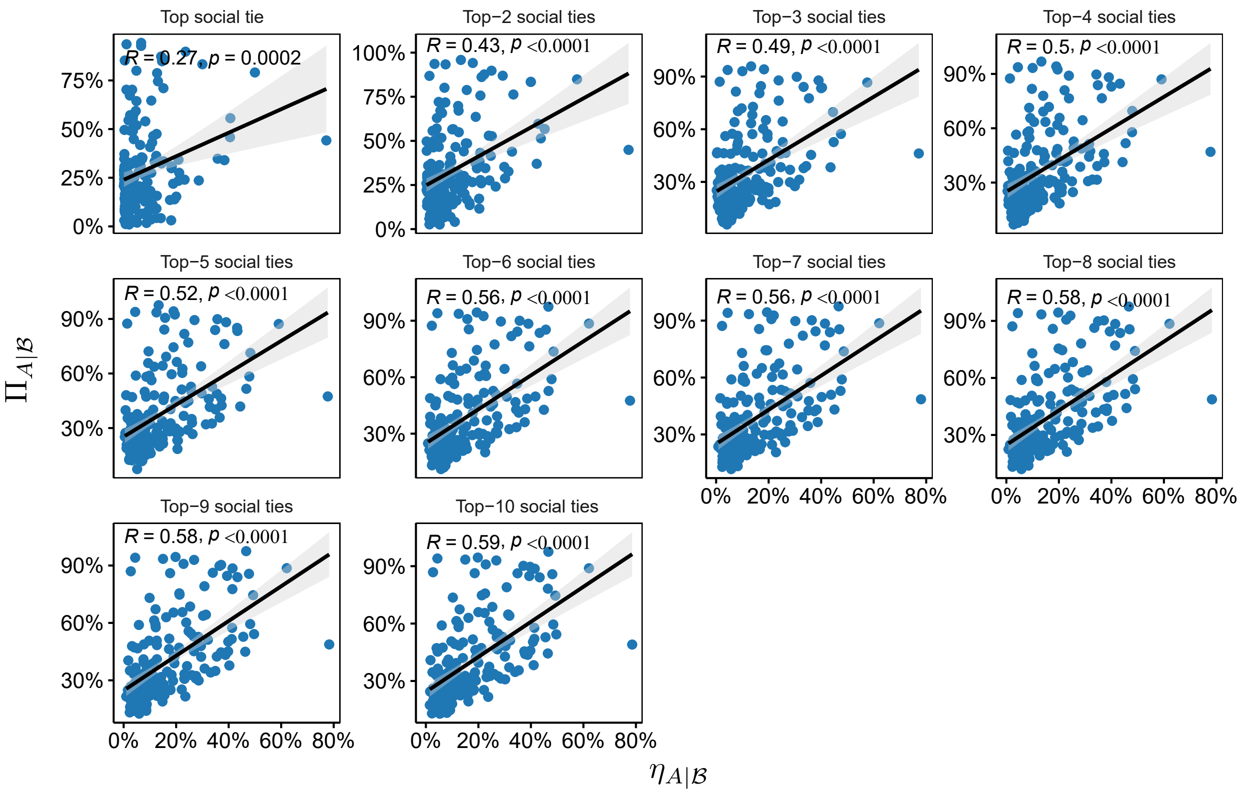

FIG. S11. **CODLR vs cumulative cross-predictability for social ties in BrightKite.** R is Pearson's correlation coefficient and p is p-value. The solid black lines are linear regression lines.

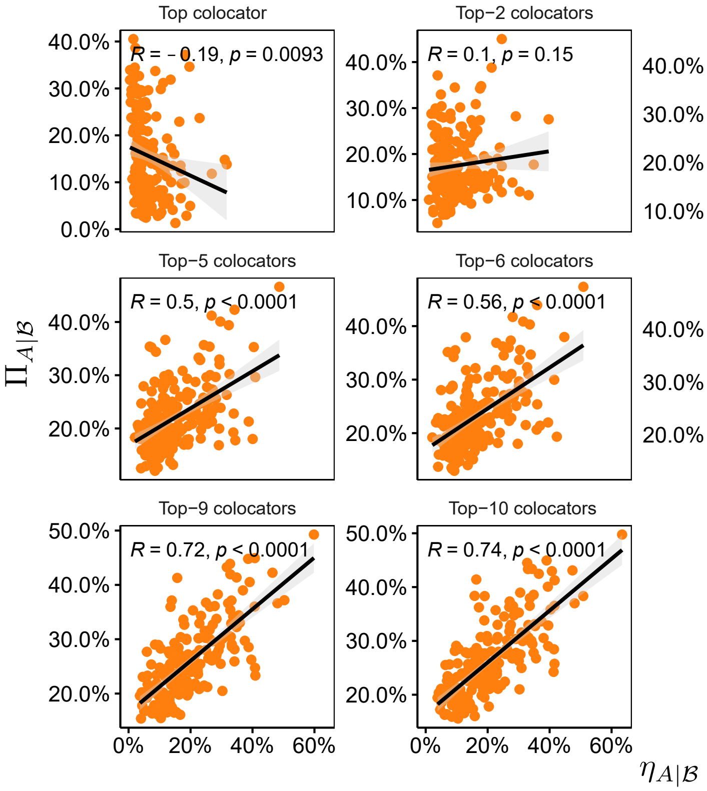

FIG. S12. **CODLR vs cumulative cross-predictability for non-social ties in Gowalla.** R is Pearson's correlation coefficient and p is p-value. The solid black lines are linear regression lines.

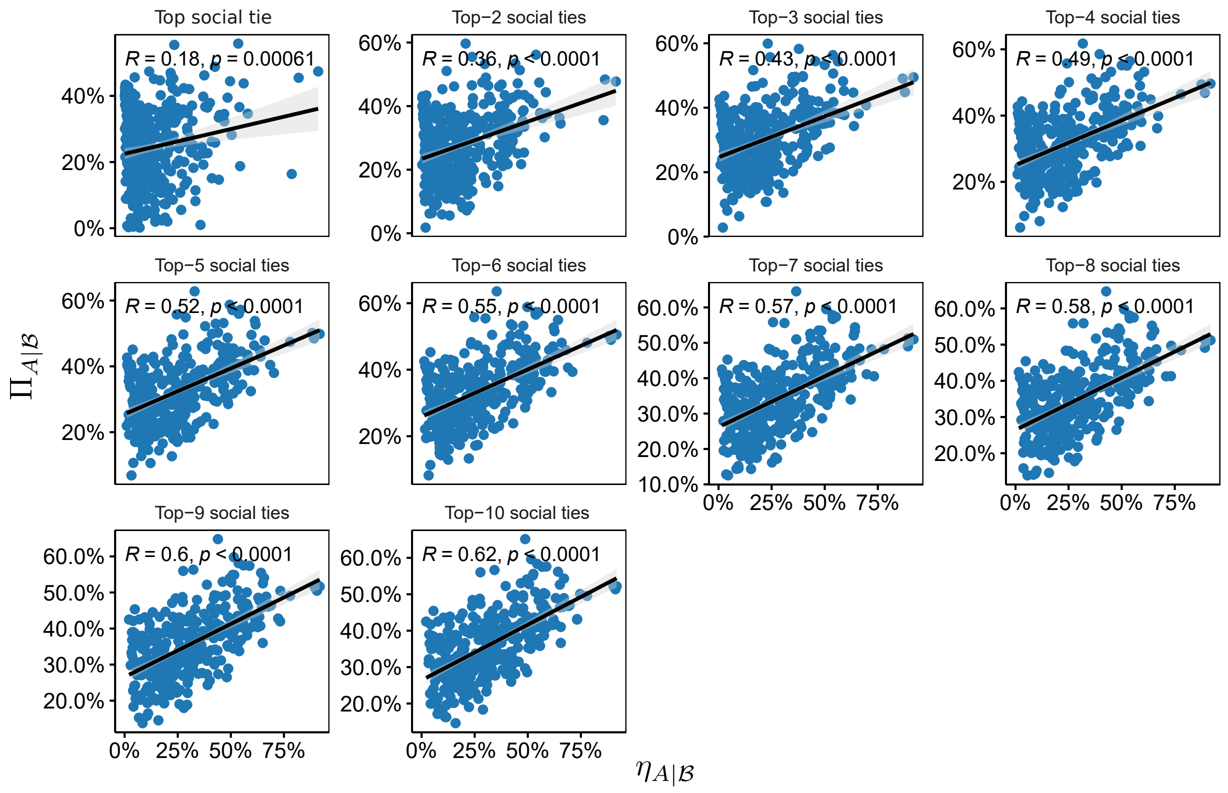

FIG. S13. **CODLR vs cumulative cross-predictability for social ties in Gowalla.** R is Pearson's correlation coefficient and p is p-value. The solid black lines are linear regression lines.

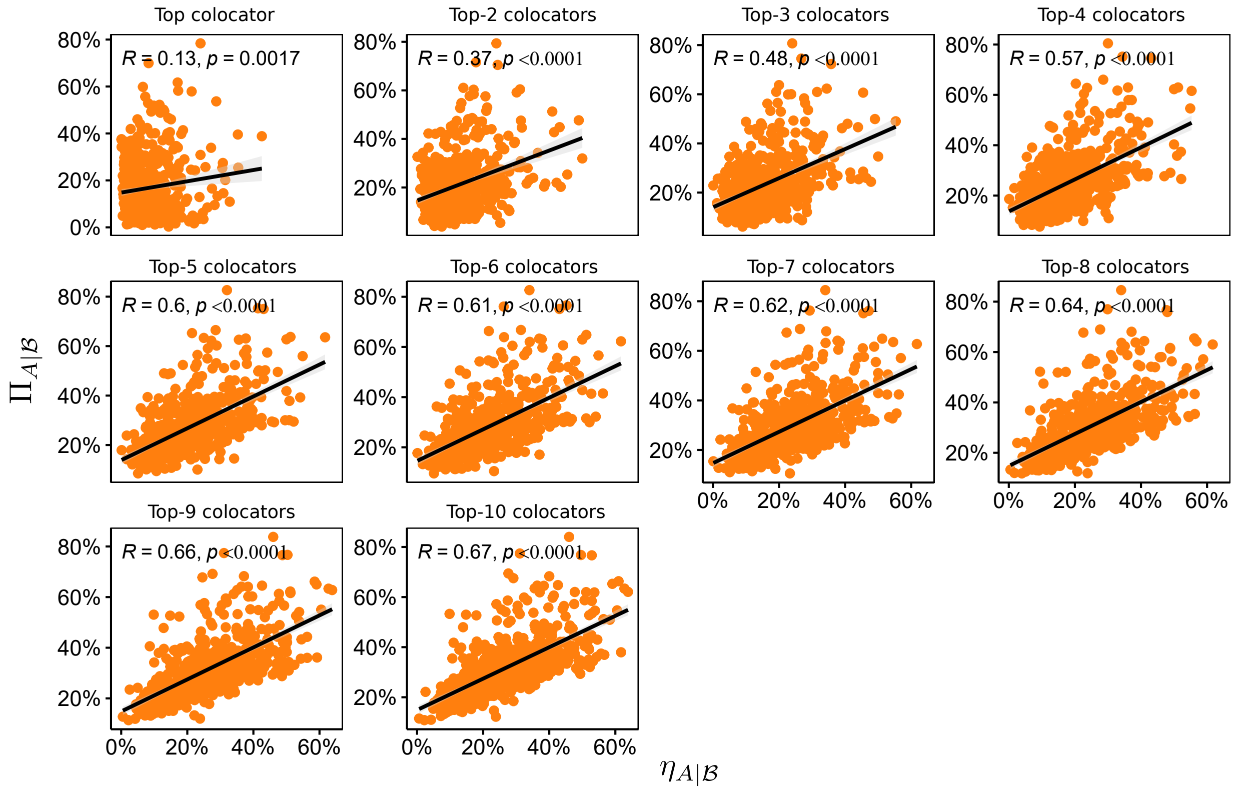

FIG. S14. **CODLR vs cumulative cross-predictability for non-social ties in Weeplaces.** R is Pearson's correlation coefficient and p is p-value. The solid black lines are linear regression lines.

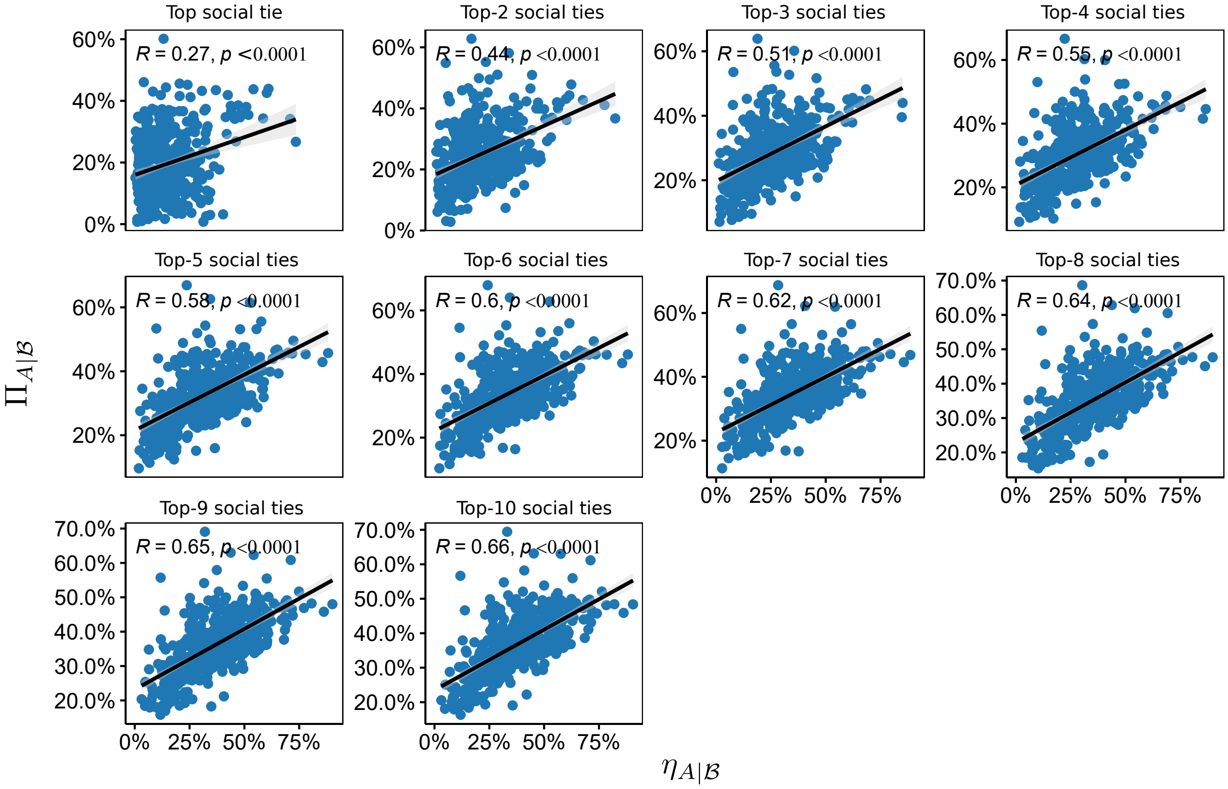

FIG. S15. **CODLR vs cumulative cross-predictability for social ties in Weeplaces.** R is Pearson's correlation coefficient and p is p-value. The solid black lines are linear regression lines.

S5.2. Correlation in CDR

We also plot the correlation between the cumulative cross-predictability and the CODLR in both types of networks as one progressively adds alters from rank-1 to rank-10 in Figure S16 and Figure S17 for the Mobile Phone dataset. As a CDR dataset, Mobile Phone dataset has lower spatial resolution and user has averagely less unique placeID compared with LBSN (See Table S1) and thus there is no clear correlation between cumulative cross-predictability and CODLR despite the increasing number of added alters.

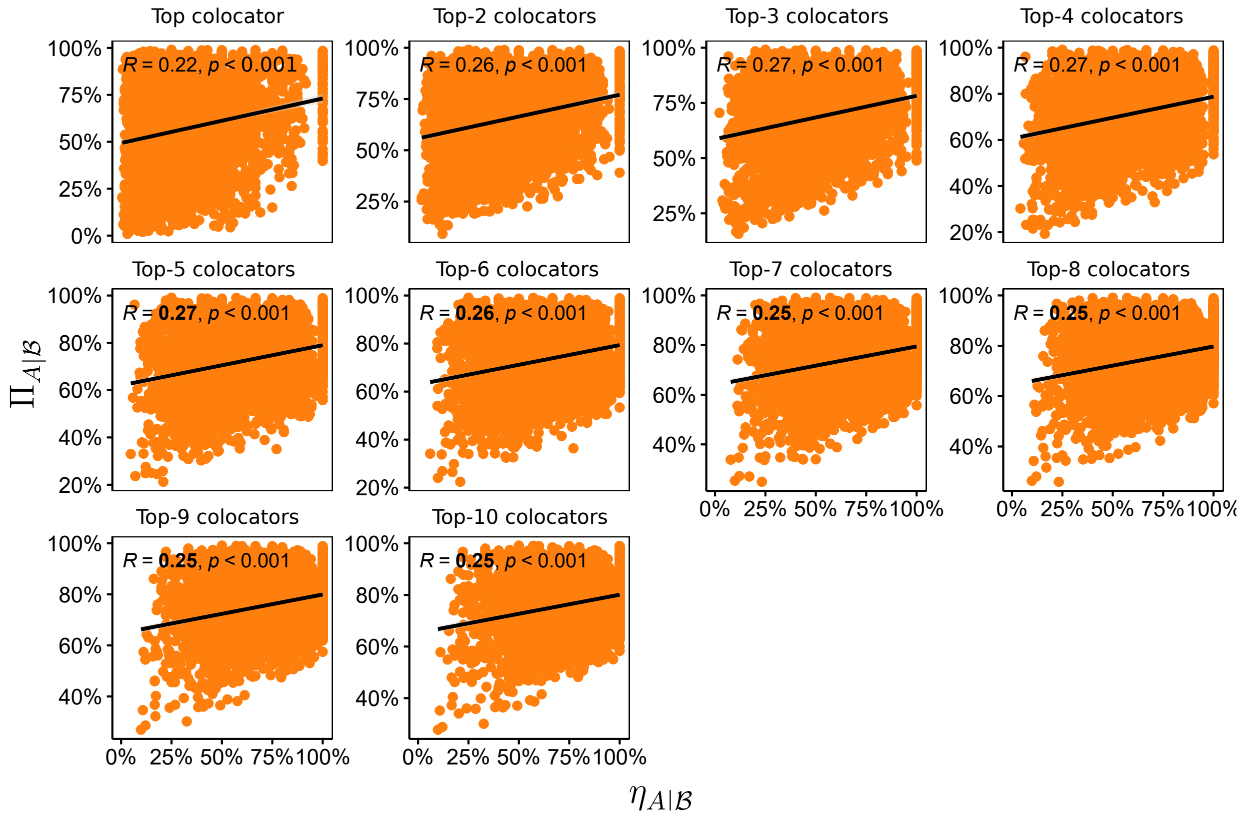

FIG. S16. **CODLR vs cumulative cross-predictability for non-social ties in Mobile Phone dataset.** R is Pearson's correlation coefficient and p is p-value. The solid black lines are linear regression lines.

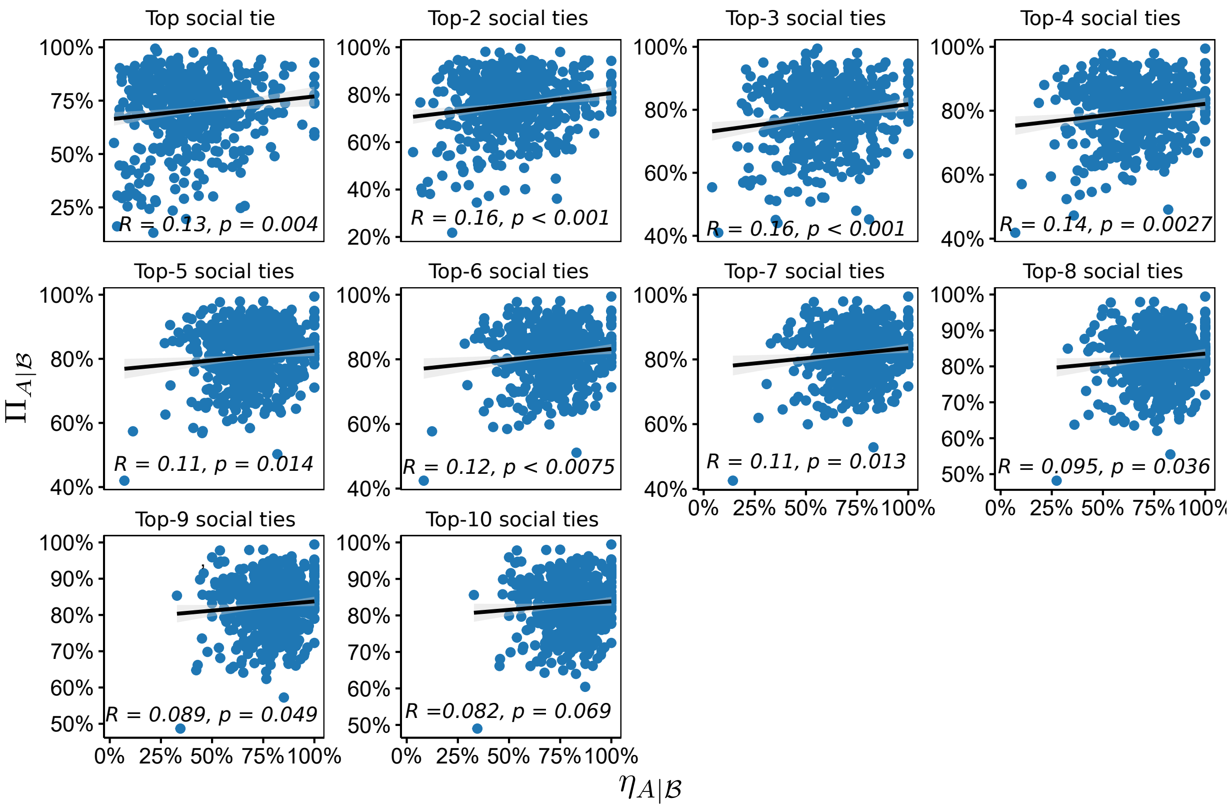

FIG. S17. **CODLR vs cumulative cross-predictability for social ties in Mobile Phone dataset.** R is Pearson's correlation coefficient and p is p-value. The solid black lines are linear regression lines.

S6. TIME LAG EFFECT

To check the similarity in pair-wise connections of the different temporal-lag networks, we compute and Jaccard similarity defined for any two sets A, B as $J(A, B) = \frac{|A \cap B|}{|A \cup B|}$, where $|\cdot|$ is the number of elements in the set. All ego-alter pairs in each type of network are considered the sets A and B.

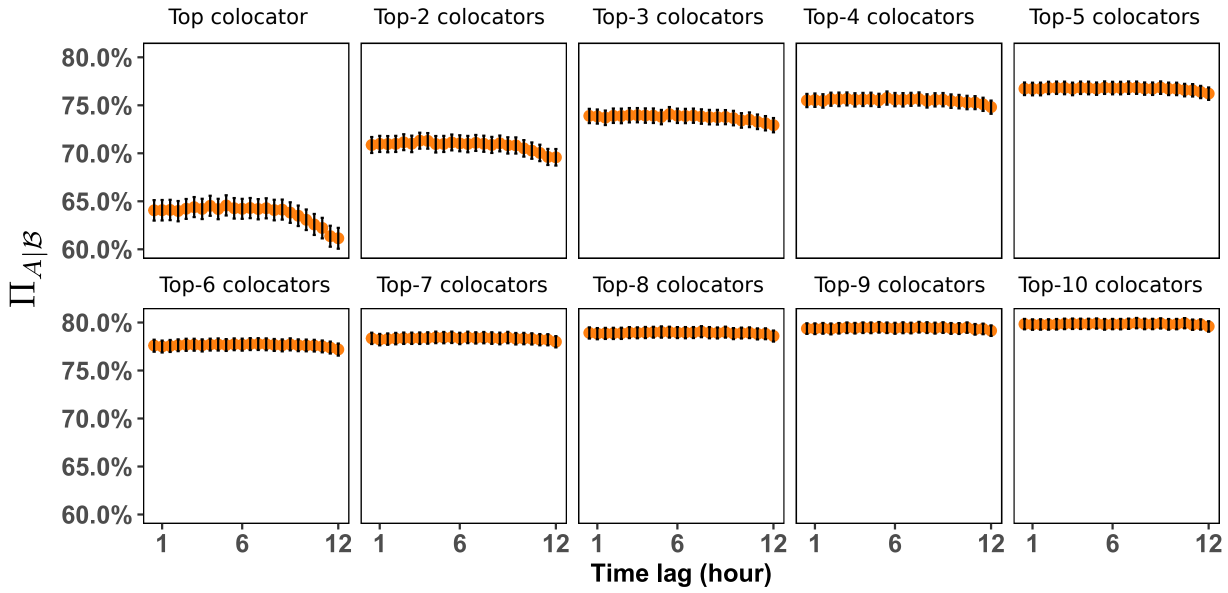

FIG. S18. **Time lag effect of non-social colocator(s) information**. The (cumulative) cross-predictability $\Pi_{A|\mathcal{B}}$ influence of temporal-lag for non-social colocator(s) in the Mobile Phone dataset. Each point corresponds to a co-location network resulting from the amount of temporal offset between an ego and alters visit to a common location. Error bars denote 95% CI.

S6.1. Sensitivity in LBSN

The results for selected temporal-lag ($T = 0.5, 3, 6, 12$) networks of Weeplaces dataset are presented in Figure S19. The T Hr-lag networks correspond to sliding windows where an ego check-in at time t colocates with an alter on the interval $(t - T, t - (T - .5)) \bigcup ((t + (T - .5), t + T)$. This means a .5 Hr-lag network corresponds to a 1 Hr sliding window colocation network with no temporal lag. The ego-alter pairs of all temporal-lag networks are generally different, but provide similar trends in cross-predictability and in cumulative

overlapped distinct locations.

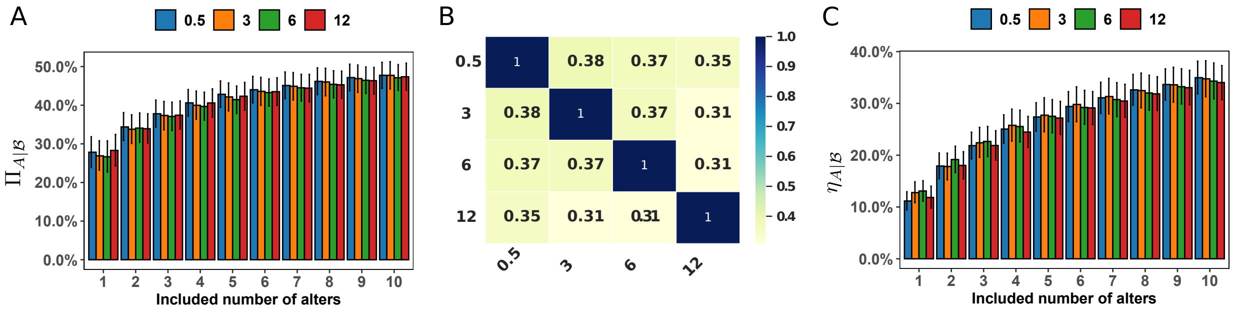

FIG. S19. **The comparison among the non-social co-located alters within 0.5H, 3H, 6H, 12H one hour sliding windows in Weeplaces dataset**. A, (Cumulative) cross-predictability $\Pi_{A|\mathcal{B}}$ versus different included number of alters. **B**, Global Jaccard Similarity between non-social co-located alters within 0.5H, 3H, 6H, 12H one hour sliding windows. **C**, (Cumulative) overlapped distinct location ratio $\eta_{A|\mathcal{B}}$ versus different included number of alters. Error bars denote mean ±95% CI.

S6.2. Sensitivity in CDR

The results for selected temporal-lag ($T = 0.5, 3, 6, 12$) networks of Mobile Phone dataset are presented in Figure S20. The full results of $\Pi_{A|\mathcal{B}}$ as a function of the temporal-lag T in Mobile Phone dataset is shown in Figure S18.

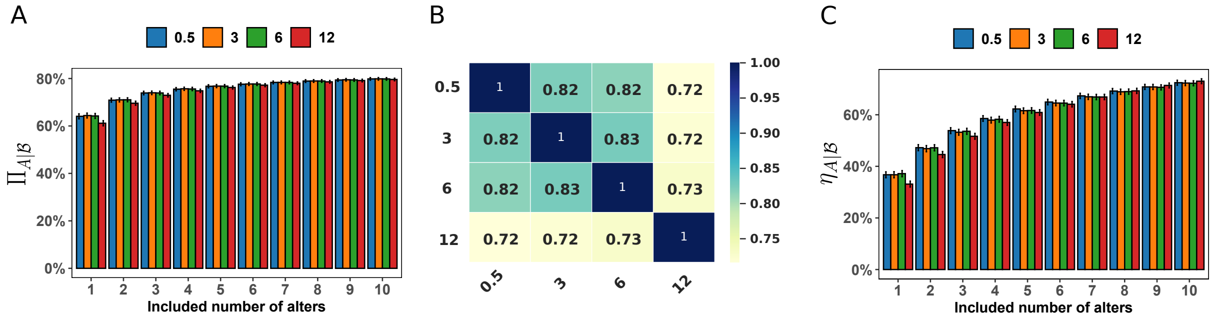

FIG. S20. **The comparison among the non-social co-located alters within 0.5H, 3H, 6H, 12H one hour sliding windows in Mobile Phone dataset**. A, (Cumulative) cross-predictability $\Pi_{A|\mathcal{B}}$ versus different included number of alters. **B**, Global Jaccard Similarity between non-social co-located alters within 0.5H, 3H, 6H, 12H one hour sliding windows. **C**, (Cumulative) overlapped distinct location ratio $\eta_{A|\mathcal{B}}$ versus different included number of alters. Error bars denote mean 95% CI.

S7. ROBUSTNESS AND CONTROLS ANALYSIS

Here we check the robustness of our results to the various filtering criteria applied to the datasets. In all cases, we measure the cumulative cross-predictability.

S7.1. Robustness of the threshold for the minimum number of check-ins

To check the sensitivity of our results to the criteria of 150 check-ins, we relax the condition to 75 check-ins. The results are plotted in Figure S21, for Weeplaces and Mobile Phone datasets. We find very little difference in both quantities (within error-bars) in either the LBSN or CDR data.

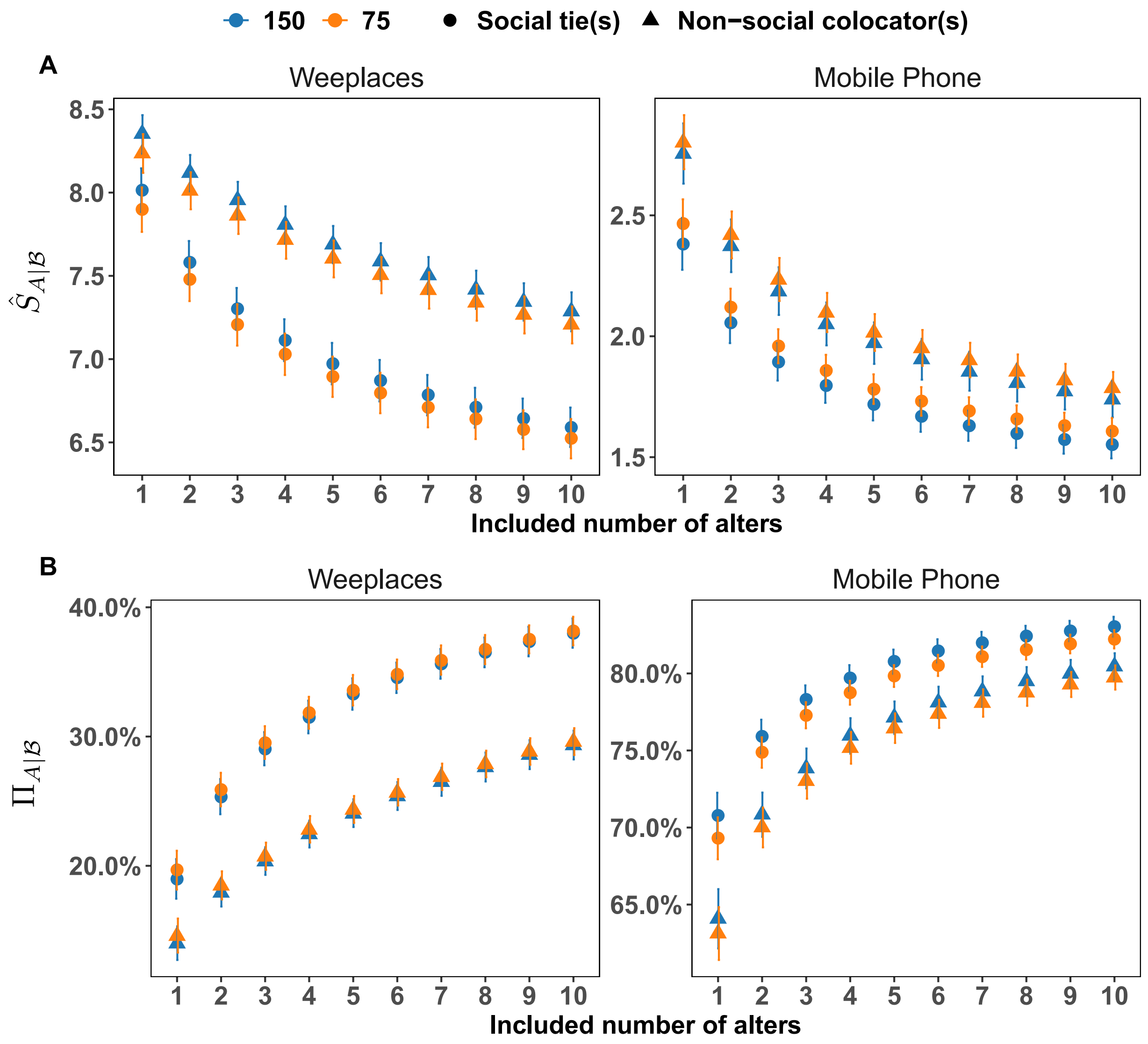

FIG. S21. **The comparison between minimum threshold settings (150 and 75) in social network and non-social colocation network from Weeplaces dataset and Mobile Phone dataset. (A)** The (cumulative) cross-entropy of egos in Weeplaces dataset and Mobile Phone dataset given by top-10 alters. **(B)** The (cumulative) cross-predictability of egos in Weeplaces dataset and Mobile Phone dataset given by top-10 alters.

S7.2. Robustness of temporal windows in colocation network construction

We check whether altering the temporal frame of co-location affect the trends in any way. To test the robustness of the 1-hour colocation time frame, we compared a 1-hour clock-bin network (colocation on a given day within the interval (T:00:00,T:59:59), $T \in (0, 1, 2..., 23)$)

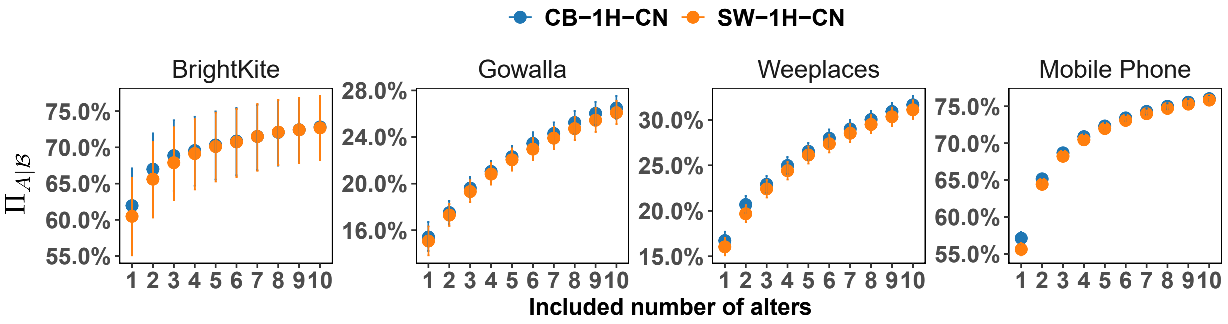

FIG. S22. **The comparison between choice of 1-hour clock-bin and sliding window in BrightKite, Gowalla, Mobile Phone and Weeplaces**. Error bars denote ±95% CI.

to a 1-hour sliding-window network (colocation within ±30 minutes of a check-in of the ego). In all four datasets Figure S22 indicates that the different temporal frames (that correspond to in general different co-location networks) have the same trends.

S7.3. Robustness to excluding "non-informative" alters

Next, we relax the condition of only including "informative" alters and include the set of all alters that have co-located more than once, whether they are "informative" or not. The resulting networks are much bigger leading to 827 and 1103 egos who have at least 10 alters in Weeplaces and Mobile Phone dataset respectively (compare to Table S2).

We plot the cumulative cross-predictabilities for Weeplaces in Figure S23A and Mobile Phone dataset in Figure S23B. For the case of Weeplaces, while we see an about a 10% drop in $\Pi_{A|\mathcal{B}}$ for both the social and non-social ties as compared to Figure 2F the qualitative trends remain robust. That is, as alters are accumulated, we see a corresponding gain on information in the ego. We find the same result for Mobile Phone dataset (panel B) with now a more modest drop of 5% in $\Pi_{A|\mathcal{B}}$. Nevertheless, once again the monotonically increasing trend of the cross-predictability via alter accumulation remains robust.

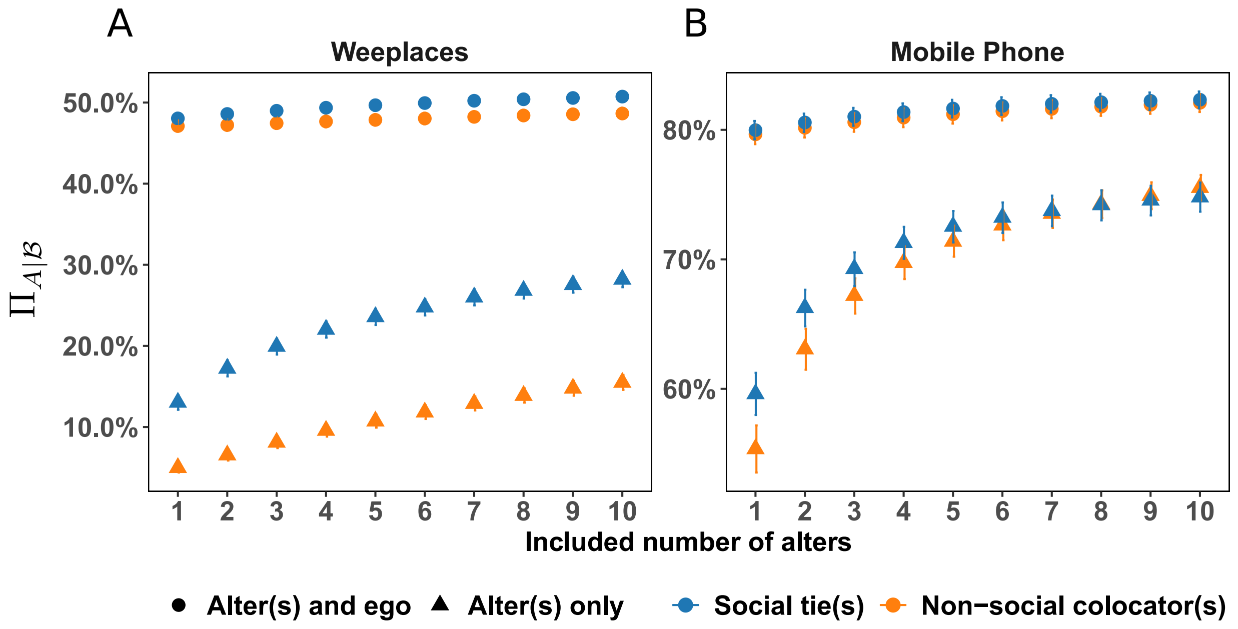

FIG. S23. **The cumulative cross-predictability including the full set of alters**. Here we include the full set of unfiltered alters ("informative" and non-informative") and construct the corresponding co-location networks, resulting in 827 egos in Weeplaces and 1103 in the Mobile Phone dataset. The resulting $\Pi_{A|\mathcal{B}}$ are for **(A)** Weeplaces dataset and **(B)** Mobile Phone dataset. Shapes indicate whether the past trajectory of the ego was included in the sequence (circles) or excluded (triangles). Error bars denote 95% CI.

S7.4. Location-overlap preserving controls

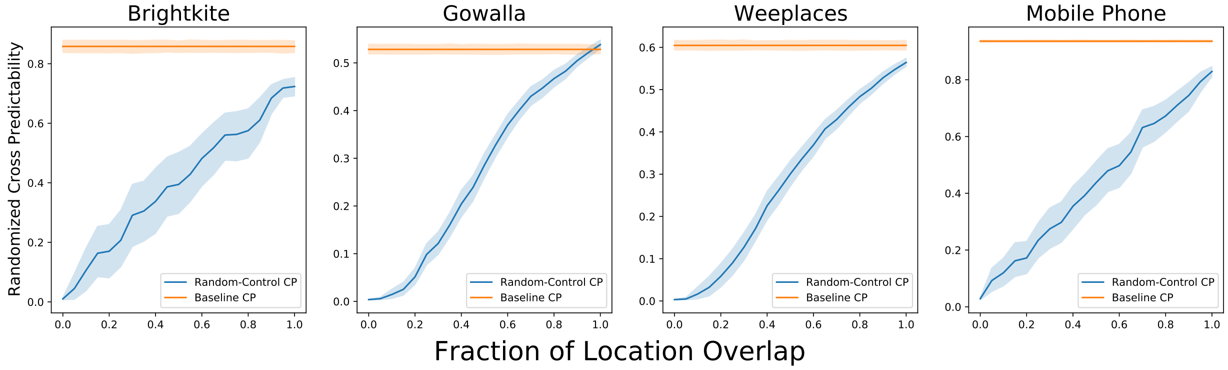

FIG. S24. **Controlling for location-overlap while randomizing sequence** The orange line refers to the average predictability over the top 20% most predictable egos in each dataset. The blue curves correspond to the cross-predictability via randomizing the original sequence and progressively decreasing location-overlap. Shaded region denotes ±95% CI.

We next check the extent to which cross-predictability of ego's can be explained entirely by its degree of location overlap with its alter. To do so, we pick the top 20% most predictable egos for each of our datasets and represent the average of their self-predictability as the horizontal orange lines in Fig. S24 . For each ego we duplicate its sequence and consider this its (perfect) alter with 100% location overlap. The sequence is then randomized a 100 times (preserving the location overlap, while destroying the time-ordering of the sequence) and an average cross-predictability is computed over those samples. Next we replace a fraction of the locations in the synthetic alter trajectory with those not present in the ego's sequence and repeat the calculation, progressively reducing the location overlap. This corresponds to the blue curves in Fig. S24. As the figure suggests, across datasets, even with 100% location overlap, there is information loss when the sequence is randomized, and that this information loss decreases monotonically with reduced location overlap.

Beyond this proof-of-concept, one can check for this effect in the empirical ego-alter trajectories. Given an ego A we randomize the trajectory (100 realizations) of each of its top 10-ranked alters $\mathcal{B}$, generating a distribution with a mean cross-predictability $\langle \Pi(A|\mathcal{B}) \rangle$. One can then formulate a hypothesis test whether the actual cross-predictability

$\Pi(A|\mathcal{B}) > \langle \Pi(A|\mathcal{B}\rangle$ at multiple levels of significance α. The results of a one-sided t-test for $\alpha = 1\%$ and 5% is shown in Fig. S25. As the figure indicates, across all datasets and for both types of networks this is true for at least 50% of egos across its top-ranked alters, with the number being between $75 - 90\%$ in BrightKite and the Mobile Phone dataset. Thus, the observed trends reported in the manuscript cannot be explained merely by location-overlap.

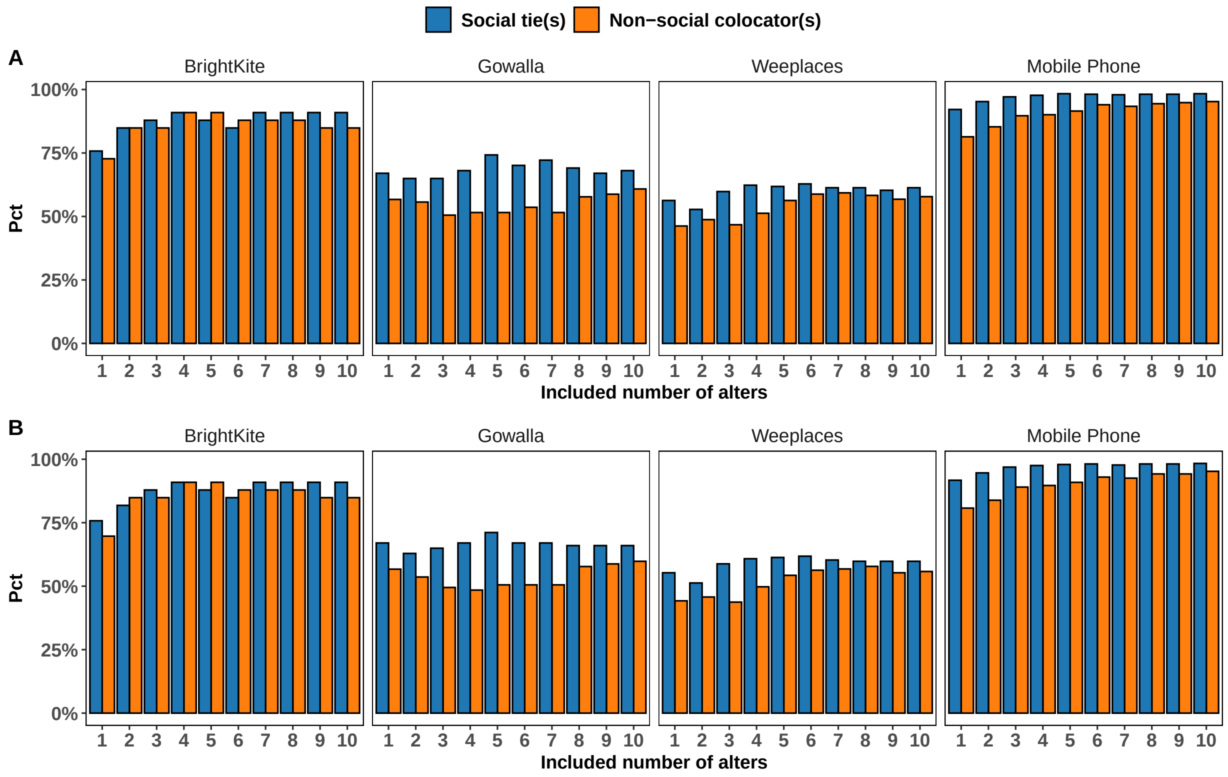

FIG. S25. **Statistical significance test for information provided by trajectory sequence** The percentage of egos for whom the cross-predictability provided by its alters exceeds that of a randomized sequence (preserving location control) at multiple levels of significance α. (**A**) $\alpha = 5\%$; (**B**) $\alpha = 1\%$.

[1] Hunter, J. D. Matplotlib: A 2d graphics environment. *Computing in Science Engineering* **9**, 90–95 (2007).
