# Supplementary material for: Contrasting social and non-social sources of predictability in human mobility
Source: Nat Commun. 2022 Apr 8;13:1922. doi: 10.1038/s41467-022-29592-y (PMC8993859; doi:10.1038/s41467-022-29592-y)
Supplement: Supplementary file 3 — Reporting Summary [file 41467_2022_29592_MOESM3_ESM.pdf]

Corresponding author(s): *Double-blind peer review submissions: write DBPR and your manuscript number here instead of author names.*

Last updated by author(s): *YYYY-MM-DD*

Reporting Summary

Nature Portfolio wishes to improve the reproducibility of the work that we publish. This form provides structure for consistency and transparency in reporting. For further information on Nature Portfolio policies, see our Editorial Policies and the Editorial Policy Checklist.

Statistics

For all statistical analyses, confirm that the following items are present in the figure legend, table legend, main text, or Methods section.

n/a	Confirmed	
☐	☒	The exact sample size (*n*) for each experimental group/condition, given as a discrete number and unit of measurement
☐	☒	A statement on whether measurements were taken from distinct samples or whether the same sample was measured repeatedly
☐	☒	The statistical test(s) used AND whether they are one- or two-sided<br>*Only common tests should be described solely by name; describe more complex techniques in the Methods section.*
☐	☒	A description of all covariates tested
☐	☒	A description of any assumptions or corrections, such as tests of normality and adjustment for multiple comparisons
☐	☒	A full description of the statistical parameters including central tendency (e.g. means) or other basic estimates (e.g. regression coefficient) AND variation (e.g. standard deviation) or associated estimates of uncertainty (e.g. confidence intervals)
☐	☒	For null hypothesis testing, the test statistic (e.g. F, t, r) with confidence intervals, effect sizes, degrees of freedom and P value noted<br>*Give P values as exact values whenever suitable.*
☒	☐	For Bayesian analysis, information on the choice of priors and Markov chain Monte Carlo settings
☒	☐	For hierarchical and complex designs, identification of the appropriate level for tests and full reporting of outcomes
☐	☒	Estimates of effect sizes (e.g. Cohen's d, Pearson's r), indicating how they were calculated

Our web collection on statistics for biologists contains articles on many of the points above.

Software and code

Policy information about availability of computer code

Data collection	The data is collected by Python 3.6 and basic library.
Data analysis	The data analysis is done by Python 3.6 and basic libraries. The link to the code can be found: https://github.com/Magica-Chen/co-locationship. The visualization is done by Python 3.6 as well as R 4.0.3 and their basic libraries.

For manuscripts utilizing custom algorithms or software that are central to the research but not yet described in published literature, software must be made available to editors and reviewers. We strongly encourage code deposition in a community repository (e.g. GitHub). See the Nature Portfolio guidelines for submitting code & software for further information.

Data

Policy information about availability of data

All manuscripts must include a data availability statement. This statement should provide the following information, where applicable:

- Accession codes, unique identifiers, or web links for publicly available datasets
- A description of any restrictions on data availability
- For clinical datasets or third party data, please ensure that the statement adheres to our policy

All analyzed data anlayzed, with the exception of the Call Data Records, is open-source for which the links have been provided within the manuscript. The CDR may be made available by the authors upon request

Field-specific reporting

Please select the one below that is the best fit for your research. If you are not sure, read the appropriate sections before making your selection.

☒ Life sciences ☐ Behavioural & social sciences ☐ Ecological, evolutionary & environmental sciences

For a reference copy of the document with all sections, see nature.com/documents/nr-reporting-summary-flat.pdf

Life sciences study design

All studies must disclose on these points even when the disclosure is negative.

Sample size	There are four datasets we considered, BrightKite, Gowalla, Weeplaces, and Mobile Phone. The first is BrightKite containing 4,491,143 geo-tagged check-ins by 58,228 users over a period of Apr 2008 - Oct 2010. The second dataset is Weeplaces, containing 7,658,368 geo-tagged check-ins produced by 15,799 users from Nov. 2003–Jun. 2011. We also include analysis on Gowalla consisting of 6,442,890 check-ins by 196,591 users over a period of Feb. 2009–Oct. 2010. Finally, we consider Mobile Phone dataset consisting of 22,116,252 call records by 35,338 users pinging 1,835 cell antennas over the period of Jan. 2014–Jun. 2014.<br>More details can be found in Supplementary Material.<br><br>All these datasets are large enough to make further analysis.
Data exclusions	We exclude any check-in records with missing attributes, e.g., time, placeid or userid. This is because these attributes have to be used in our analysis.<br>We also establish a 150 check-in threshold that yields a sufficient amount of data to analyze the both the individual user entropy and cross entropy,<br>More details can be found in Supplementary Material.
Replication	All analysis as well as results can be reproduced.
Randomization	No samples/organisms/participants were allocated into experimental groups because our study do not apply any random algorithms to making experimental groups.
Blinding	*Describe whether the investigators were blinded to group allocation during data collection and/or analysis. If blinding was not possible, describe why OR explain why blinding was not relevant to your study.*

Reporting for specific materials, systems and methods

We require information from authors about some types of materials, experimental systems and methods used in many studies. Here, indicate whether each material, system or method listed is relevant to your study. If you are not sure if a list item applies to your research, read the appropriate section before selecting a response.

Materials & experimental systems

n/a	Involved in the study	
☒	☐	Antibodies
☒	☐	Eukaryotic cell lines
☒	☐	Palaeontology and archaeology
☒	☐	Animals and other organisms
☒	☐	Human research participants
☒	☐	Clinical data
☒	☐	Dual use research of concern

Methods

n/a	Involved in the study	
☒	☐	ChIP-seq
☒	☐	Flow cytometry
☒	☐	MRI-based neuroimaging
